# The Evolution of the Musculoskeletal Trauma Section of the Orthopaedic In-Training Examination

**DOI:** 10.5435/JAAOSGlobal-D-22-00184

**Published:** 2023-04-12

**Authors:** M. Kareem Shaath, Christopher H. Garrett, Jianna Lin, Frank R. Avilucea, Mark W. Munro, Joshua R. Langford, George J. Haidukewych

**Affiliations:** From the Orlando Health Jewett Orthopedic Institute, Florida State College of Medicine, University of Central Florida College of Medicine, Orlando, FL (Dr. Shaath, Dr. Avilucea, Dr. Munro, Dr. Langford, and Dr. Haidukewych); the Orlando Health Jewett Orthopedic Institute (Dr. Garrett); and the University of Florida College of Medicine Ms. Lin).

## Abstract

In 1963, the American Academy of Orthopaedic Surgeons administered the Orthopaedic In-Training Examination (OITE), the first and longest running yearly medical specialty examination. There have been no recent studies to evaluate the content of the musculoskeletal trauma section of the OITE.

**Methods:**

We analyzed all questions that were classified by the American Academy of Orthopaedic Surgeons as musculoskeletal trauma from 2012 to 2019. We recorded the number of musculoskeletal trauma questions in each examination, the topics and imaging modalities tested, the references cited, and the taxonomy classification of each question. We extrapolated from a similar musculoskeletal trauma study published in 2011 to create the previous examination cohort for comparison.

**Results:**

For the current cohort, the average number of musculoskeletal trauma questions was 43.5 questions per examination (18.4%). The most frequently tested topics were proximal tibia fractures, pediatric trauma, hip fractures, and diaphyseal femur fractures, respectively. In previous examinations, questions from T1 and 2 were tested significantly more frequently compared with the current examinations (*P* < 0.001 and *P* = 0.02, respectively). In the current cohort, T3 questions were tested significantly more frequently than previous examinations (*P* = 0.001). Previous examinations had significantly more questions without an image (36 questions per year versus 25 questions per year, *P* < 0.001). In current versions of the examination, radiographs are tested significantly more frequently than other imaging modalities (*P* < 0.001).

**Discussion:**

The musculoskeletal trauma section of the OITE has evolved. To improve and focus study efforts, residents may use this study as a guide when preparing for the examination.

In 1963, the American Academy of Orthopaedic Surgeons (AAOS) administered the Orthopaedic In-Training Examination (OITE), the first and longest running yearly medical specialty examination.^1^ The examination was the idea of then AAOS president elect, Dr. J. Vernon Luck, in 1960.^2^ The goals of the examination were to provide an examination that will permit the chiefs of training programs to evaluate the quality, scope, and comprehensiveness of their educational systems; to provide information to the residents who take the examination as to their performance in relation to their peer groups; to provide an instrument which may be used as a valuable education aid; and to investigate new methods of testing in education.^2^ The original examination consisted of 150 multiple choice questions while modern versions of the examination contain 275 multiple choice questions in 12 categories: foot and ankle, hand, orthopaedic science, hip and knee reconstruction, orthopaedic diseases, spine, pediatric orthopaedics, medical-related issues, sports medicine, musculoskeletal trauma, rehabilitation, and shoulder and elbow.^1,2^ Historically, the examination was administered on paper; however in 2009, the examination became computer-based.^3^ Multiple studies have demonstrated that resident OITE performance correlates with success on the (American Board of Orthopedic Surgery) Part I examination.^3,4,5,6^

With the goal of improving resident performance in the OITE, multiple studies have detailed OITE subspecialty-specific content.^1,2,7,8,9,10,11,12,13,14^ OITE content undergoes frequent modifications to adhere to the advancing field of orthopaedics, and recent studies have been conducted to determine whether subspecialty content in the OITE changes over time; however, this has not been done for the musculoskeletal trauma section.^15,16^ Our goal for this study was to analyze OITE musculoskeletal trauma questions from 2012 to 2019 and compare them with the results reported by Cross et al and Lackey et al from 2011.

## Methods

We analyzed all questions that were classified by the AAOS as musculoskeletal trauma from 2012 to 2019. From 2012 to 2016, electronic versions of the examination with answers and explanations were available for download. From 2017 onward, the examinations with answers and explanations could only be accessed through ResStudy offered by the AAOS.^17^ We recorded the number of musculoskeletal trauma questions in each examination, the topics and imaging modalities tested, the references cited, and the taxonomy classification of each question based on previously published studies.^8,10,12,18,19^ The taxonomic classifications were knowledge (taxonomy I), comprehension (taxonomy 2), and application (taxonomy 3). Taxonomy I questions require recall of facts; taxonomy 2 questions require the interpretation of information and images to make a diagnosis; and taxonomy 3 questions represent the highest levels of cognitive requirement and closely simulate the higher cognitive processes used in practice as the test taker is required to formulate a treatment plan.^1,13^

The results were then compared with the data of Cross et al and Lackey et al, which will be referred to as the previous examinations or the previous cohort.^1,14^ Descriptive statistics were used to evaluate the data, and a two-sample Student *t*-test was used to analyze the data between two groups. Analyses were conducted using Minitab (Minitab), and significance was set at *P* = 0.05. No outside sources of funding were used.

## Results

For the current cohort, the average number of musculoskeletal trauma questions was 43.5 questions per examination (18.4%) (range 31 to 52 questions) (Table 1). No difference was observed in the total number of questions from previous examinations (Table 1). The most frequently tested topics were proximal tibia fractures, pediatric trauma, hip fractures, and diaphyseal femur fractures, respectively. The least tested topics were pathologic fractures, spinal cord injuries, and arthrotomies. The specific topics tested are listed in Table 2.

**Table 1:** Number and Type of Musculoskeletal Trauma Question by Year

Factor	2005	2006	2007	2008	2009	2012	2013	2014	2015	2016	2017	2018	2019	*P*
Musculoskeletal trauma (or trauma)-related questions	52	52	49	50	52	52	52	50	51	50	43	62	31	0.53
Total questions	272	271	268	270	270	271	268	275	275	275	271	270	260	0.37
Percentage of trauma-related questions	19.1	19.2	18.3	18.5	19.3	19.2	19.4	18.1	18.5	18.2	15.9	23.0	11.9	—
Trauma knowledge and recall (T1)	7	7	8	14	14	16	21	23	20	22	18	23	17	**<0.001**
Diagnosis and interpretation (T2)	17	9	9	7	8	5	8	1	4	7	5	3	2	**0.02**
Treatment and decision making (T3)	17	9	9	7	8	31	23	26	27	21	20	36	11	**0.001**

**Table 2:** List of Specific Topics Offered Arranged From Most to Least Frequently Tested

Injury	2012	2013	2014	2015	2016	2017	2018	2019	Total
Proximal tibia fractures (including plateau)	5	3	4	8	5	4	1	0	30
Pediatric trauma	0	0	0	0	0	14	12	2	28
Hip fractures (including intertrochanteric and subtrochanteric fractures)	1	3	6	8	3	2	3	0	26
Diaphyseal femur fractures	4	5	2	2	6	0	1	0	20
Pelvic ring injuries	3	1	0	2	3	4	2	3	18
Ankle fractures	1	3	0	3	9	0	0	0	16
Distal tibia “pilon” fractures	3	3	4	2	2	2	0	0	16
Humeral shaft fractures	3	4	1	1		1	3	2	15
Tibial shaft fractures	3	3	3	2	1		1	1	14
Acetabular fractures	0	1	1	0	2	1	6	3	14
Implant biomechanics	0	1	4	1	2	2	2	2	14
Distal humerus fractures	2	1	2	5	1	0	1	1	13
Distal femur fractures	4	0	2	0	5	1	0	0	12
Basic anatomy	4	3	1	1	1	1	0	0	11
Antibiotic administration/infection control	1	3	3	1	2	0	0	1	11
Gunshot wounds	0	1	2	2	1	0	2	2	10
Traumatic hemorrhage/hemodynamics/shock	0	1	3	2	0	0	3	1	10
Fracture biology	0	4	2	1	2	0	0	0	9
Patellar fractures	2	1	3	1	1	1	0	0	9
Distal radius fractures	4	0	0	0	1	0	3	0	8
Nonunion	1	2	2	0	0	0	2	0	7
Foot fractures (including Lisfranc injuries)	1	3	0	1	0	1	0	1	7
Nerve injury	0	0	0	0	0	1	3	3	7
Polytrauma	2	1	1	0	1	1	0	0	6
Patellar or knee dislocation	0	2	1	0	1	1	0	1	6
Periprosthetic fractures	0	0	0	0	0	2	4	0	6
Intimate partner violence (IPV)/nonaccidental trauma	0	0	1	1	0	1	1	1	5
Compartment syndrome	0	0	0	1	0	0	3	1	5
Clavicle fractures	1	0	0	1	0	1	0	1	4
Tendon/ligament injuries	1	0	0	0	0	1	1	0	3
Sentinel event errors	0	1	1	1	0	0	0	0	3
Proximal humerus fractures	0	0	0	0	0	0	2	1	3
Forearm fractures	0	0	0	1	1	0	0	0	2
Scapula fractures	0	0	0	1	0	0	0	1	2
Heterotopic ossification	1	0	0	0	0	0	0	1	2
Supracondylar femur fractures	0	2	0	0	0	0	0	0	2
Osteomyelitis	2	0	0	0	0	0	0	0	2
Olecranon fractures	1	0	0	1	0	0	0	0	2
Vascular injuries	0	0	0	0	0	0	1	1	2
Elbow/terrible triad injuries	0	0	0	0	0	0	2	0	2
Talar fractures/dislocations	1	0	0	0	0	0	0	0	1
Hand fractures	1	0	0	0	0	0	1	0	2
Femoral valgus deformity	0	0	1	0	0	0	0	0	1
Implicit bias (cultural competence)	0	0	0	1	0	0	0	0	1
Pathologic fractures	0	0	0	0	0	0	1	0	1
Spinal cord injuries	0	0	0	0	0	0	1	0	1
Arthrotomies	0	0	0	0	0	1	0	0	1

In previous examinations, knowledge and recall (T1) questions were the most frequently tested, followed by diagnosis and interpretation (T2) and treatment and decision-making (T3) questions, respectively. In the current examination cohort, T3 questions were the most frequently tested, followed by T1 and 2 questions, respectively. In previous examinations, questions from T1 and 2 were tested significantly more frequently compared with the current examinations (*P* < 0.001 and *P* = 0.02, respectively) (Table 1). In the current cohort, T3 questions were tested significantly more frequently than previous examinations (*P* = 0.001) (Table 1).

*The Journal of Orthopaedic Trauma* was the most frequently cited journal in both cohorts, followed by *The Journal of Bone and Joint Surgery* and *The Journal of the Academy Orthopaedic Surgeons* (Table 3). The journal entitled *Orthopedics* was significantly cited more frequently in questions from the current cohort compared with the previous cohort (*P* = 0.01). *The Journal of Orthopedic Trauma* was cited significantly more frequently than *The Journal of Bone and Joint Surgery* and *The Journal of the Academy Orthopaedic Surgeons* (*P* = 0.018 and *P* = 0.007, respectively). *The Orthopaedic Knowledge Trauma Update* was the most cited textbook in the current cohort while *Rockwood and Green's Fractures in Adults* was the most cited textbook in the previous cohort (Table 4). No significant relationships were observed when textbooks were compared between cohorts, and the overall number of textbook citations were similar between cohorts (*P* = 0.18). In the current cohort, *The Journal of Orthopaedic Trauma* was cited significantly more frequently than all textbook citations combined (21.3 citations per year versus 9.5 citations per year *P* = 0.002).

**Table 3:** Top Journal Citations by Frequency

Journal	2005	2006	2007	2008	2009	Total	2012	2013	2014	2015	2016	2017	2018	2019	Total	*P*	Average Age of Citation (12–19) (yr)
*Journal of Orthopaedic Trauma*	26	32	18	29	21	126	16	18	17	15	31	27	23	23	170	0.26	3.2
*Journal of Bone and Joint Surgery*	17	18	34	19	21	109	13	12	27	19	10	12	15	12	120	0.18	3.6
*Journal of the Academy Of Orthopaedic Surgeons*	4	5	8	13	7	37	9	16	12	13	9	6	28	3	96	0.17	2.8
*Journal of Pediatric Orthopedics*	0	0	0	0	0	0	2	3	0	4	2	19	11	3	44	0.05	3.0
*Clinical Orthopaedics and Related Research*	11	13	5	6	7	42	3	4	5	11	3	6	2	4	38	0.09	3.7
*Injury*	2	2	3	2	3	12	2	2	0	5	4	4	12	5	34	0.19	3.0
*Journal of Trauma*	9	5	8	3	3	28	6	7	2	2	6	2	4	2	31	0.27	4.3
*Bone and Joint Journal*	1	4	5	3	6	19	4	5	5	5	5	1	4	0	29	0.88	3.2
*Foot and Ankle International*	0	5	0	2	1	8	3	2	1	2	3	0	2	2	15	0.79	3.2
*Orthopedics*	0	0	0	0	0	0	2	1	2	5	2	1	1	1	15	**0.01**	3.1
*Instructional Course Lectures*	2	1	1	0	4	8	2	1	1	0	2	3	3	0	12	0.94	2.9
*Journal of Shoulder and Elbow Surgery*	1	0	0	3	0	4	2	0	0	3	0	0	4	2	11	0.50	3.5
*Journal of Trauma and Acute Care Surgery*	0	0	0	0	0	0	0	0	0	0	1	3	4	1	9	0.15	3.1
*American Journal Of Sports Medicine*	0	3	0	0	0	3	3	1	3	0	1	0	0	0	8	0.61	3.7
*Archives of Orthopedics and Trauma Surgery*	0	0	2	0	1	3	3	1	0	0	2	0	0	1	7	0.64	3.4

**Table 4:** Textbook Citations in Order of Frequency

Textbook	2005	2006	2007	2008	2009	Total	2012	2013	2014	2015	2016	2017	2018	2019	Total	*P*
Orthopaedic Knowledge Update: Trauma 2/3/4	9	1	0	4	3	17	8	10	8	1	0	0	0	0	27	0.99
Orthopaedic Knowledge Update 7/9/10/11	5	0	0	2	3	10	2	3	0	4	0	0	0	0	9	0.34
Rockwood and Green's Fractures in Adults	0	0	1	7	11	19	0	0	0	1	4	0	0	1	6	0.25
Campbell's Operative Orthopaedics	0	0	0	0	0	0	0	0	0	0	6	0	0	0	6	0.50
Skeletal Trauma: Basic Science, Management, and Reconstruction	0	5	8	8	1	22	2	0	0	2	0	0	0	0	4	0.09
Principles of Deformity Correction	0	0	0	0	0	0	1	2	0	0	0	0	0	0	3	0.61
Fractures of the Pelvis and Acetabulum	0	0	0	0	0	0	1	0	0	0	0	1	2	0	4	0.31
AO Principles of Fracture Management	0	0	0	3	1	4	0	0	0	0	1	1	0	1	3	0.53
Fractures of the Acetabulum	2	1	0	0	5	8	1	0	0	0	0	0	0	0	1	0.14
Other textbooks	0	0	0	0	0	0	5	1	0	4	2	0	1	0	13	**0.04**
Total	16	7	9	24	24	80	20	16	10	12	13	0	3	2	76	0.18

Questions without associated images were the majority in both cohorts (Table 5). In previous examinations, there were significantly more questions without an image compared with current examinations (36 questions per year versus 25 questions per year, *P* < 0.001). Current examinations offer more groups of imaging combinations than previous examinations (10 versus 3), and the current cohort had significantly more questions with an associated image than the previous cohort (24 questions per year versus 14 questions per year, *P* = 0.003). In current versions of the examination, radiographs are tested significantly more frequently than other imaging modalities (*P* < 0.001). No significant relationships were found when comparing specific imaging modalities (Table 5).

**Table 5:** Tested Imaging Modalities

Imaging Modality Tested	2005	2006	2007	2008	2009	Average	2012	2013	2014	2015	2016	2017	2018	2019	Average	*P*
No image	36	40	37	32	37	36.4	29	28	29	25	26	18	24	19	24.8	**<0.001**
Radiograph	14	10	9	16	11	12	15	17	18	18	14	20	34	10	18.3	0.05
CT	1	1	1	1	0	0.8	1	1	1	4	2	1	1	0	1.4	0.12
Radiograph & CT	0	1	2	1	4	1.6	4	3	2	2	2	3	2	2	2.5	0.14
MRI	0	0	0	0	0	0	1	0	0	1	0	0	0	0	0.3	0.43
CT & MRI	0	0	0	0	0	0	0	0	0	0	0	1	0	0	0.1	>0.05
Radiograph & MRI	0	0	0	0	0	0	0	0	0	0	0	0	1	0	0.1	>0.05
Photograph	0	0	0	0	0	0	0	3	0	0	3	0	0	0	0.8	0.16
Photograph & MRI	0	0	0	0	0	0	1	0	0	0	0	0	0	0	0.1	>0.05
Photograph & radiograph	0	0	0	0	0	0	1	0	0	1	3	0	0	0	0.6	0.25
Total with images	15	12	12	18	15	14.4	23	24	21	26	24	25	38	12	24.1	**0.003**
Total trauma questions	51	52	49	50	52	50.8	52	52	50	51	50	43	62	31	48.9	0.28
Percentage with images	29.4	23.1	24.5	36.0	28.8	28.3	44.2	46.2	42.0	51.0	48.0	58.1	61.3	38.7	49.3	—

## Discussion

One of the major goals of orthopaedic residency programs is to ensure their graduates pass the American Board of Orthopedic Surgery examinations.^3^ In addition, to maintain residency program accreditation, a board certification rate of at least 75% is required.^3^ It has been demonstrated that OITE performance correlates with success on American Board of Orthopedic Surgery Part I examination.^3,4,5,6^ Therefore, it is in the best interest of both orthopaedic surgery residents and residency programs to perform well in this examination. The purpose of our study was to provide an in-depth analysis of the musculoskeletal trauma section of the OITE while comparing it with prior versions of the examination with the ultimate goal to improve resident performance in this section of the examination.^1,14^

Historically, the musculoskeletal trauma section comprises nearly 20% of the OITE and is larger than other content areas tested, and improving scores in this section may have the largest effect in improving residents' overall performance.^1^ To improve resident performance, we have included all question topics tested in this section over an 8-year period. When preparing for the examination, residents may use Table 2 to focus their study efforts. Prior studies have listed previously tested topics in the examination; however, the lists were not as specific or exhaustive.^1,14^ It should be noted that certain question topics such as pathologic fractures, hand fractures, and spinal cord injuries may also appear in other subspecialty sections.

Although the quantity of musculoskeletal trauma questions was similar over the years, their content was not. Previous examinations focused on simpler questions revolving around recall and diagnosis, with a minority dedicated to treatment and decision making. Other OITE subspecialty analyses over a similar period as the previous examination cohort reported recall-focused questions to be the most prevalent as well.^1,7,8,10,11,12,13,14^ Nearly half (49.9%) of the questions in recent versions of the examination focus on treatment and decision making, representing the highest levels of cognitive requirement and closely simulate the higher cognitive processes used in practice.^1,13^ Other OITE subspecialty analyses over a similar period as the current examination cohort report a shift toward decision-making and evaluation questions as we did.^16,20^ When preparing for the examination, residents should focus their efforts on how to apply their knowledge in patient care revolving around treatment and decision making.

Imaging was included on the OITE in 1968.^21^ Previous examinations relied on questions that did not require the interpretation of imaging while recent versions of the examination had significantly more questions that required the interpretation of at least one image, comprising nearly half of the questions (49.3%). Newer versions of the examination also had more combinations of different imaging modalities as well. Schulz et al^16^ reported similar findings as well when analyzing the oncology section.

Many journals include articles on musculoskeletal trauma, but when studying for the OITE, residents should focus their efforts on *The Journal of Orthopaedic Trauma*, *The Journal of Bone and Joint Surgery*, and *The Journal of the Academy of Orthopaedic Surgeons. The Journal of Orthopedic Trauma* was cited markedly more frequently than any other journal in the musculoskeletal trauma section. Although the authors of this study think that textbooks are a valuable learning resource for residents, they should choose either *Skeletal Trauma* (Browner et al, Saunders) or *Rockwood and Green's Fractures in Adults* (Bucholz et al, Lippincott Williams and Wilkins) as a resource, but not both.^14^ While preparing for the musculoskeletal portion of the OITE, residents must keep in mind that *The Journal of Orthopedic Trauma* alone was cited markedly more frequently than all textbooks combined in the current cohort. Given that the average age of citation is from 2.8 to 3.2 years, residents would be best served by reviewing articles from this range when preparing for the examination.

There are a few differences of our study when compared with the studies by Cross et al and Lackey et al. An important distinction between our analyses is the OITE became computer-based in 2009.^1,3,14^ All OITE examinations, except for one, in the previously referenced studies were paper-based. Given that modern iterations of the examination are computer-based, our analysis becomes critical. We have demonstrated several differences between examinations that were likely facilitated by using a computer-based examination such as more questions requiring the interpretation of images and more combinations of imaging modalities. In addition, we have included an 8-year period, including the most recent OITE, while the other studies included a 5-year period and did not include the most recent OITE examination at the time of publication.^1,14^ With a longer study period, we offer comprehensive data and can more accurately describe the evolution of the examination. Another strength of our study is that we include the longest consecutive period when analyzing a subspecialty of the OITE.

A potential limitation of this study is that the periods of our study and the previously referenced studies are discontinuous. Although we may not be able to determine the true nature of the evolution of the examination, an 8-year period is the highest reported in the literature. Another potential limitation is that none of the authors of the current study collaborated on the studies by Lackey et al and Cross et al, although we attempted to follow their methodologies as closely as possible. In addition, because question types and taxonomic levels were determined by a single institution, there is a potential for bias, although we used a system that is widely used.^8,10,12,18,19^

The musculoskeletal trauma section of the OITE has evolved. To improve and focus study efforts, residents should concentrate on how to apply their knowledge in the treatment of musculoskeletal injuries, using Table 2 as a guide to commonly tested topics. Residents should also be prepared to answer more difficult questions focusing on treatment and decision making while interpreting at least one image, most commonly a radiograph. Residents should be familiar with articles from *The Journal of Orthopaedic Trauma* and should only use one textbook as a resource. We think that implementing these strategies will lead to a better performance in the musculoskeletal trauma section and the OITE as a whole.
